# Visualising surgical training in O&G following the COVID-19 pandemic - The European view

**DOI:** 10.52054/FVVO.14.4.044

**Published:** 2023-01-27

**Authors:** R Hablase, R Mallick, F Odejinmi

**Affiliations:** Royal Sussex County Hospital, University Hospitals Sussex NHS Foundation Trust, Eastern Road, Brighton, BN25BE, UK; Princess Royal Hospital, University Hospitals Sussex NHS Foundation Trust, Lewes Road, Haywards Heath, RH16 4EX, UK; Whipps Cross Hospital, Barts Health NHS Trust, Whipps Cross Road, Leytonstone, London, E11 1NR, UK

**Keywords:** COVID-19, coronavirus, training, Europe

## Abstract

**Background & Objectives:**

Obstetrics and Gynaecology (O&G) training programmes that traditionally relied on the hands-on apprenticeship-training model, became crippled with the global response to the COVID-19 pandemic.

**Methods:**

Web-based anonymised survey was circulated to trainee members of the European Society for Gynaecological Endoscopy (ESGE) over 8-weeks period commencing June 2021.

**Results:**

213 trainees from 20 countries responded. Trainees from medium Human Development Index (HDI) countries were less represented. 78% (166/213) were in approved training programmes and 81% (174/213) had access to personal PPE. The vaccine uptake was 87% (185/213). 39% (89/213) and 55% (118/213) experienced negative impact on their physical and mental wellbeing with 36% (76/213) COVID-19 related absence. 15% (32/213) were redeployed to areas outside O&G. 25% (53 /213) had negative impact on their obstetric experience compared to 54% (114/213) reported lower gynaecology surgical exposure and 43% (91/213) failed to meet their gynaecology surgical competencies during the pandemic. 64% (137/213) perceived simulation training as an alternative training tool.

**Conclusion:**

In the post-pandemic recovery phase, gynaecological societies and national institutes across Europe continue to develop training curricula implementing virtual and hybrid training modules. The aim is to develop a robust blueprint to safeguard the gynaecological surgical training in the future.

**What is new?:**

The ongoing impact on the training in the post pandemic era remains to be evaluated. Our pan Europe survey highlights areas that remain affected from trainees’ perspective and assesses differences in the healthcare systems across continent. We then discuss the novel initiatives taken to overcome training gaps.

## Background

The unexpected COVID -19 crisis has disrupted medical education and affected training programmes across the world (Shah et al., 2020). In the UK, the negative impact on obstetrics and gynaecology (O&G) training continues to be felt despite the restoration of normal National Health Services (NHS) activities (Mallick et al., 2020) and this is likely to be felt for many years to come. A similar negative impact on O&G trainees in Italy and in wider Europe has also been reported (Boekhorst et al., 2021). Thus, to aid recovery, comparing training trends across different health care systems following the global pandemic is crucially important to capture the ongoing challenges and the evolving landscape of surgical training delivery in O&G. This will allow positive surgical training techniques and gold standard training practice to be highlighted and create a blueprint that can be established Europe- wide to not only safeguard training in the event of future challenges, but ultimately improve training for all.

On a positive note, the pandemic appears to have created the momentum needed to modernise surgical training delivery tools (Elnikety et al., 2022). Albeit challenging, training programmes are gaining resilience and robust identification of creative training pathways are being adopted to preserve the quality of the surgical training delivered (Duggan et al., 2022).

In our study, we surveyed the European Society for Gynaecological Endoscopy (ESGE) members across Europe (and beyond) comparing responses trends to previously published data. The survey questions spanned a wide range of experiences including training opportunities, surgical practices, the ongoing impact on the mental and physical wellbeing in addition to the long-term impact of the pandemic on training and career pathways. We then discussed measures implemented by regulatory training bodies and societies to create a gynaecological surgical training pathway fit for the future.

## Methods

We sent a web-based anonymised voluntary survey to all trainee members of the ESGE over an 8-week period from the 2nd of June, 2021. The survey link was circulated via email in the English language. Participants were informed of the length of time of the survey, the purpose of the study and informed consent obtained. The IP (internet protocol) address of the client computer was used to identify potential duplicate entries from the same user. Duplicates were excluded from data analysis, with the first entry included. All incomplete questionnaires were included in the analysis irrespective of the number of questions completed. No geographical exclusions were included. The questionnaire included baseline respondent’s characteristics, training country and level, trainees’ health and safety, changes in both obstetrics and gynaecology clinical activities, redeployment, COVID-19 sickness, departmental teaching, and training delivery tools. The questionnaire was developed as a combination of positive and negative questions in a non-consequence manner to minimise potential bias. White space questions were added to invite comments and suggestions. Statistical analysis was performed using Statistical Package for Social Sciences (SPSS) v. 26.0 (IBM Inc). Data are shown as means with standard deviation or as number and percentages. Thematic analysis of the data including trainee comments was also undertaken.

## Results

A total of 213 trainees from 20 countries responded. Using the human development index (HDI) classification (a composite index of life expectancy, education and per capita income indicators used to rank countries into four tiers of human development: very high, high, medium and low) 206 respondents were from very high HDI (UK 112, Germany 64, Belgium 5, France 4, Turkey 4, Portugal 3, Switzerland 3, Netherland 2, Greece 2, Romania 2, Austria 1, Finland 1, Georgia 1, Bahrain 1, Chile 1). 2 were from high HDI (Ukraine and South Africa) and 5 were from medium HDI (India 3, Sri Lanka 1, Pakistan 1).

In total 78% (166/213) were in approved training programme in their country of training. 58% (123/213) had more than 6 years of formal O&G training with 46% (99/213) had 3 years or less left to complete their training at the onset of the pandemic. The demographics of the respondents are summarised in Table I.

**Table I:** Respondents demographics.

Demographic	% (n)
Age	
25 to 34	82 (39)
35 to 44	76 (36)
45 to 54	30 (14)
55 to 64	20(9)
Over 65	5(2)
Gender	
Male	71 (33)
Female	141 (66)
Prefer not to say	1(1)
Ethnicity	
White	143(67)
Mixed	9 (4)
Asian	30 (14)
Black	21 (10)
Any other ethnic group	4 (2)
Prefer not to disclose	6(3)
Marital Status	
Married	125(59)
Single	53(25)
Civil partnership	11(5)
Co-habiting	22(10)
Prefer not to say	2(1)

### Health and wellbeing

Access to personal protective equipment (PPE) had improved from 60% (129/213) at the onset of the pandemic to 81% (174/213) currently. However, only 54% (115/213) received appropriate training on the use of PPE at the onset of the pandemic. 36% (76/213) took time off related to COVID-19 and 20% (42/213) had previously had COVID- 19. The mean number of days taken off due to COVID-19 was 18 days (SD± 24). The uptake of the vaccine was 87% (185/213) among the ESGE trainee members. 39% (89/213) and 55% (118/213) agreed that the pandemic had negative impact on their physical and mental wellbeing respectively. 33% (70/213) had support systems in place through their training programme.

### Work and training

15% (32/213) were redeployed to cover areas outside O&G. Of those, 7% (14/213) felt clinically competent to do so. 25% (53 /213) agreed or strongly agreed that the COVID-19 pandemic negatively impacted their obstetric training experience. 24% (56/213) felt a negative impact on their obstetrics antenatal experience, while 35% (75/213) reported a negative impact on their obstetric ultrasound training experience.

**Figure 1:**
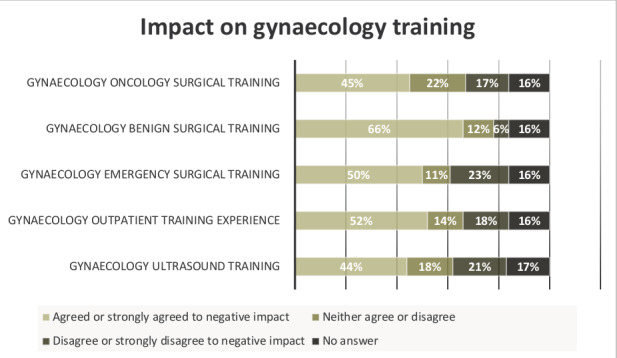
Impact on gynaecology training.

The negative impact on various aspects of gynaecological training was more profound. Figure 1 summarises the perceived impact.

Overall, 54% (114/213) reported a lower or significantly lower gynaecological surgical exposure compared to pre-pandemic times. 43% (91/213) had not met their gynaecological surgical training competencies during the pandemic.

### Long Term impact and career pathway

**Figure 2:**
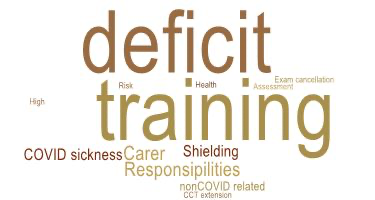
Reasons for training extension.

66% (140/213) agreed or strongly agreed that their training had been negatively impacted for the 12 months following the onset of the pandemic. 41% (87/213) had experienced a negative impact on their annual appraisal / yearly training outcome. 24% (51 /214) reported that COVID-19 has resulted in an unwanted, but necessary change in their career/ training plans. 15% (31/213) had their training extended due to COVID-19. The mean extension period was 6 months (SD±2.9). Reasons for training extension varied and are summarised in Figure 2.

### Training and Education

**Figure 3:**
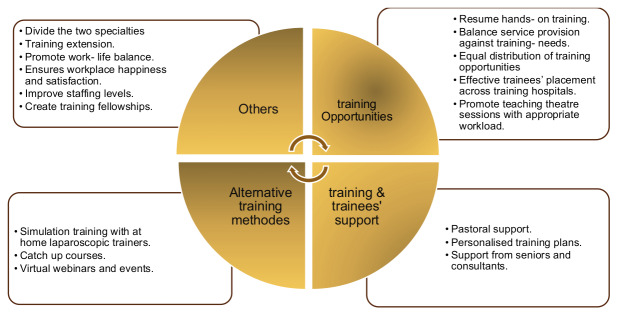
Trainee responses to ‘how they could best be supported to minimise the impact of the pandemic moving forward.

Only 16% (35/213) felt that operating in the independent (private) sector may represent a feasible alternative to gaining surgical skills whilst 27% (57/213) positively perceived simulation training to aquire gynaecological surgical competencies. 24% (51/213) were able to meet their gynecological competencies required for their stage of training. Slightly above two-thirds of the trainees reported a negative impact on their educational activities. Webinars and virtual training opportunities were largely percieved as a positive outcome of the pandemic amongst 64 % (137/213) of the ESGE trainee members. However 44% (94 /213) reported a negative impact on work-life balance of conducting webinars and virtual training out of hours. In general 65% (140/213) were optimistic that O&G training would recover from the negative impact of the pandemic. Figure 3 summarises the main themes when trainees were asked to comment on how they could best be supported to minimise the impact of the pandemic moving forward.

**Table II:** ESGE members responses regarding wellbeing, training, and future.

	Strongly agree/agree (%)	Neither agree nor disagree (%)	Strongly disagree/disagree (%)
The COVID-19 pandemic has affected your physical wellbeing.	39	19	26
The COVID-19 pandemic has affected your mental wellbeing	55	15	14
COVID-19 has negatively affected your obstetric clinical training experience.	25	20	39
COVID-19 has negatively affected your obstetric antenatal training experience.	26	20	38
COVID-19 has negatively affected your obstetric ultrasound experience.	35	24	24
COVID-19 has negatively affected your gynaecological ultrasound experience.	45	17	21
COVID-19 has negatively affected your gynaecological outpatient training experience.	52	14	17
COVID-19 has negatively affected your emergency gynaecological surgical training experience.	45	11	22
COVID-19 has negatively affected your benign gynaecological surgical training experience.	67	5	12
COVID-19 has negatively affected your gynaecological oncological surgical training experience.	45	22	17
I have met my gynaecological surgical training competencies during the pandemic.	24	17	42
My educational opportunities have been negatively affected by COVID-19.	62	12	9
COVID-19 had a negative impact on my yearly training outcome /ARCP	41		43
COVID-19 resulted in an unwanted, but necessary change in my career/training plans.	24	20	35
Operating in the independent/ private sector has been a feasible alternative to gaining surgical competencies.	16	20	47
Simulation training has been a feasible alternative to gaining gynaecological surgical competencies.	27	22	35

## Discussion

Since COVID-19 was declared a pandemic by the World Health Organization (WHO) on March 11, 2020 (Mallick et al., 2021), unprecedented changes to health care systems have occurred and continue to evolve. France, Germany, UK, Spain, and Italy were the top five European countries with the highest numbers of COVID-19 cases (Pang et al., 2020). In all these five countries and subsequently other European countries, major elective surgeries were cancelled, telemedicine implemented, and work force restructured to cope with the unexpected crisis (COVIDSurg collaborative, 2020). In the aftermath of the pandemic the ramifications of these measures on the postgraduate training in obstetrics and gynaecology (O&G) remains to be fully addressed (Ostapenko et al., 2020) and it is likely the impact will be felt for many years to come.

Despite the heterogeneity of O&G trainees’ activities in different health care systems across Europe, existing studies have highlighted the negative impact of the pandemic on the mental and physical wellbeing of the trainees. A survey from UK and Italy showed that more than half of the respondents (60% and 58% respectively) experienced a negative psychological impact (Bitonti et al., 2020; Duggan et al., 2022). Similarly, in our study the results were comparable at 55%.

The redeployment rates in our survey were 15%, which is comparable to those reported at the onset of the pandemic between April and July 2020 (Boekhorst et al., 2021). This may suggest that following the initial redeployment there has been little or no further redeployment of trainees.

24% of our respondents had agreed or strongly agreed to the negative impact of COVID-19 pandemic on their obstetrics and antenatal training, which is comparable to the UK survey at 22% (Duggan et al., 2022). There was a general perception among the European trainees that obstetric care during the pandemic had not been affected (Boekhorst et al., 2021). This may explain the less negative impact on the obstetrics training in general.

On the contrast, there had been a significant reduction in surgical training across all specialties. Hence, the impairment had been more profound on gynaecology training in general (Bitonti et al., 2020). Despite the restoration of near normal activities in many healthcare systems, trainees continued to experience a reduction in learning opportunities in gynaecology (Mallick et al., 2021). In our survey, 45%, 50% and 66% reported a negative impact on their oncology, emergency, and benign gynaecology surgical skills, respectively.

The reduction in elective surgery was in the interest of patient safety and supporting the wider response. However, cancelling elective surgery on such a wide scale has had a substantial impact on patients and cumulative, potentially devastating, consequences for healthcare systems and training programmes. (COVIDSurg collaborative, 2020). It is estimated that during the 12-week peak of the pandemic 726 822 gynaecological surgeries were cancelled in Europe and central Asia, 90% of which were for “benign” gynaecology disease (Ball et al., 2021). Hence, it is not surprising that 54% of the trainees in our study reported a reduction in the exposure to gynaecology surgeries with 43% unable to meet their yearly training competency targets.

The slow recovery of gynaecology surgical training had also been highlighted in an earlier survey from Europe (Boekhorst et al., 2021) and a follow-up survey from the UK (Duggan et al., 2022). Recovery from the backlog created during the first wave of COVID-19 with a 20% increase in the baseline surgical volume was estimated to take countries a median of 45 (range 43–48) weeks to clear. This did not account for the further disruption created by the second and third waves of COVID-19.

The strains of the surgical list back log, re- configuration of patients’ pathways and staff shortage due to sickness continue to pressurise the already challenged training system across Europe. In addition, adoption of conservative approaches to certain pathologies, changes to multidisciplinary management (MDM), dual consultant operating and prioritisation of procedures across specialties have added further challenges (COVID-STAR collaborative, 2021).

It is becoming evident that closing the gynaecological surgical training gaps will require shifting the training platform to at least partially virtual. The concept of home surgical training is evolving, and an abundance of resources are being utilised including home trainers, virtual reality simulators, webinars, video games and even hobbies (Hoopes et al., 2020). Training bodies and societies across the continent continue to work on developing a structured framework of such resources within O&G training programmes.

Simulation training is not new and modern curricula across Europe have been promoting a simulation component in combination with the clinical experience (Zimmerman et al., 2021). However, implementation varies significantly across the European countries. In the UK, for example, the 2019 trainee evaluation survey highlighted that less than 80% of O&G trainees had access to a structured simulation programme. It is undoubtedly that simulation and virtual training platforms have gained the momentum during the pandemic and will continue to evolve as a fundamental training delivery tool. The European Board and College of Obstetrics and Gynaecology (EBCOG) in their “Project for Achieving Consensus in Training (PACT),” published in 2018, recommended simulation training to be used to support both core and subspecialty skills acquisition. Core skills simulation training includes ultrasound, diagnostic and therapeutic hysteroscopy, and laparoscopy, suturing techniques, and coil placement. Subspeciality skills may include, for example, embryo transfer. Similarly, the statements from Health Education England (HEE, 2021) and the recently published Training in gynaecological surgery recovery plan (RCOG, 2021) promoted the implementation of structured national simulation programmes.

Surgical simulators have been proven to shorten clinical learning curves, and it was demonstrated that techniques learnt by using a simulator can be brought into the operation room (Weiss and Rentea, 2022). In the UK, the British Society for Gynaecological Endoscopy (BSGE) has recently initiated a structured centralised programme delivered through a series of hands-on workshops combined with online webinars and take-home training box, to alleviate training gaps.

The European Society for Gynaecological Endoscopy (ESGE) runs The Gynaecological Endoscopic Surgical Education and Assessment (GESEA) programme. The programme follows the European–American Joint Recommendation stating that each hospital teaching endoscopic surgery should make available an endoscopic dry lab for training and improving the proficiency of the endoscopic surgery skills of the physician. The programme provides participants with the knowledge and psychomotor skills necessary to enter the operating room or to start supervised basic endoscopic procedures. Furthermore, it offers advanced masterclasses, workshops, and clinical immersions through a combination of hands-on workshops and virtual webinars. (Bustos et al., 2020).

Promoting participation in such structured multimodal programme is encouraged particularly in the post COVID-19 era where there is a transition to alternative methodology in obtaining gynaecology surgical skills (McMurray et al., 2022).

Facilitating trainees’ mobility across borders through agreed travelling fellowships or exchange programme for all or part of the training period needs to be considered on the continent level. This may not necessarily be a sustainable or a wide scale measure to close the training gaps following the pandemic, however it remains an appealing idea on multiple levels and a similar programme had been implemented by the European Society of Gynaecological Oncology (ESGO). Furthermore, national training programmes should be encouraged to be more flexible to allow trainees to take time out of their training programmes to travel elsewhere in their country or beyond to gain the surgical skills they may not have the opportunity to develop during standard training.

In the context of published literature our study strength comes from the timeline at which it was conducted, further away from the peak of the pandemic and into the recovery phase, reflecting the ongoing training struggles and projected on the training recovery. The study had also compared training trends across a larger cohort of the European trainees. We acknowledge that there may have been an element of selection bias due to the differential availability of technological infrastructure used to circulate the survey. This may explain the underrepresentation of medium and low HDI countries. We also acknowledge that the variable response rate across the European countries makes it difficult to establish the training struggles in every European country with precision particularly with most of the respondents from UK and Germany. It is difficult of establish if those struggling more with surgical training opportunities were more likely to respond and share their experiences and whether trainees in countries with lower response rates have had a better training recovery with more surgical training opportunities than others, hence we must accept that there is an element of bias. In addition, the respondents were ESGE members with variable age range and training status. The survey aimed to emphasise the subjective perception of training difficulties from a trainee’s perspective.

The long-term impact of the pandemic in terms of full training recovery and positive implementation of new training delivery modalities remain to be evaluated in the future research.

## Conclusions

O&G training programmes across Europe are continuing to struggle with surgical training gaps. Obstetrics training has or almost has recovered to the pre-pandemic baseline, whilst gynaecological surgical training has had a much slower pace of recovery. National training bodies and societies are developing and implementing virtual platforms, simulation, and hybrid training delivery modules. These implementations were COVID-19 born and are in the early developmental stages, however they need to be fully explored and engrained into gynaecological surgical training to safeguard it in the future. A true Europe wide approach with targeted and structured surgical training programmes and integrated simulation training utilising different types of training modalities shared across geographical boundaries are the key to success in what appears to be a new era for medical education.
